# Co-Administration of Lactulose Crystals with Amoxicillin Followed by Prolonged Lactulose Treatment Promotes Recovery of the Human Gut Microbiome In Vitro

**DOI:** 10.3390/antibiotics11070962

**Published:** 2022-07-18

**Authors:** Cindy Duysburgh, Pieter Van den Abbeele, Dennis Franckenstein, Martin Westphal, Angelika Kuchinka-Koch, Massimo Marzorati

**Affiliations:** 1 ProDigest BV, Technologiepark 82, 9052 Ghent, Belgium; Cindy.Duysburgh@prodigest.eu (C.D.); pieter.vandenabbeele@cryptobiotix.eu (P.V.d.A.); 2 Fresenius-Kabi Deutschland GmbH, Else-Kröner-Str. 1, 64352 Bad Homburg, Germany; dennis.franckenstein@fresenius-kabi.com (D.F.); martin.westphal@fresenius-kabi.com (M.W.); 3 Fresenius-Kabi Austria GmbH, Estermannstrasse 17, 4020 Linz, Austria; angelika.kuchinka-koch@fresenius-kabi.com; 4 Center of Microbial Ecology and Technology (CMET), Ghent University, Coupure Links 653, 9000 Ghent, Belgium

**Keywords:** *Bifidobacterium*, *Lactobacillus*, SHIME, antibiotic-associated diarrhoea, dysbiosis, clavulanic acid

## Abstract

The validated SHIME model was used to assess the effect of repeated administration of two different lactulose dosages (5 g/d and 10 g/d) on the human gut microbiome during and following amoxicillin–clavulanic acid treatment. First, antibiotic treatment strongly decreased *Bifidobacteriaceae* levels from 54.4% to 0.6% and from 23.8% to 2.3% in the simulated proximal and distal colon, respectively, coinciding with a marked reduction in butyrate concentrations. Treatment with lactulose enhanced acetate and lactate levels during antibiotic treatment, likely through lactulose fermentation by *Lachnospiraceae* and *Lactobacillaceae*. One week after cessation of antibiotic treatment, *Bifidobacteriaceae* levels re-increased to 20.4% and 7.6% in the proximal and distal colon of the 5 g lactulose/d co-administered unit, as compared with 1.0% and 2.2% in the antibiotic-treated unit, and were even further stimulated upon extension of lactulose administration. Marked butyrogenic effects were observed upon prolonged lactulose supplementation, suggesting the establishment of cross-feeding interactions between *Bifidobacteriaceae* and butyrate producers. Furthermore, a limited *Enterobacteriaceae* outgrowth following antibiotic treatment was observed upon dosing with 10 g lactulose/d, indicating inhibition of pathogenic colonization by lactulose following antibiotic therapy. Overall, lactulose seems to be an interesting candidate for limiting the detrimental effects of amoxicillin–clavulanic acid on the human gut microbiome, though further studies are warranted to confirm these findings.

## 1. Introduction

Penicillin-class antibiotics are used to treat a wide variety of bacterial infections. While being effective towards killing disease-causing agents, they also cause collateral damage by adverse effects on indigenous microbes, such as health-related *Bifidobacterium* and *Lactobacillus* species [1]. The resulting dysbiosis of the gut microbiome has been linked to gastrointestinal disorders, such as diarrhoea, most often caused by amoxicillin [2]. There is thus a need to develop strategies preventing or at least limiting microbial dysbiosis caused by antibiotics.

One of the strategies to possibly improve human health through modulation of the gut microbiome considers supplementation with prebiotics, which are defined as non-digestible substrates that selectively stimulate specific gut microbes, consequently conferring a health benefit on the host [3]. While prebiotic intake has been related to a wide range of biological effects, including immune stimulation [4,5], several studies have also reported a potential role for prebiotics in the prevention and/or treatment of non-antibiotic-associated infections by stimulation of indigenous gut microbiota [6]. With respect to the prevention of antibiotic-associated diarrhoea, studies are scarcer and mainly focus on the stimulation of *Bifidobacterium* species to counteract the creation of a dysbiosed microbial community due to antibiotic therapy [7,8,9,10,11].

Lactulose, a synthetic non-digestible disaccharide, is well-known for its therapeutic use in treating constipation [12] and hepatic encephalopathy [13]. Furthermore, several other health benefits have been reported for lactulose, including the stimulation of beneficial micro-organisms and inhibition of pathogenic bacteria along the gastrointestinal tract [14,15]. Recent in vitro studies revealed the prebiotic potential of lactulose, which was able to strongly enhance the abundance of health-promoting bifidobacteria and lactobacilli, as well as several other species, including *Alistipes*, *Parabacteroides*, *Anaerostipes* [16,17], *Megasphaera* [18] and the butyrate-producing *Faecalibacterium prausnitzii* [19]. In addition, Nikolaou et al. [20] have shown that children treated with the antibiotic azithromycin in combination with lactulose showed faster recovery of intestinal homeostasis by stimulation of saccharolytic bacteria, such as *Lactobacillus*, *Anaerostipes* and *Roseburia*, providing evidence that lactulose could be an interesting compound for use in minimizing adverse effects induced by antibiotic therapy.

The aim of the current study was to evaluate the potential effect of different doses of lactulose (5 g/d and 10 g/d) in supporting the recovery of the activity and composition of the gut microbiota upon co-administration with the antibiotic amoxicillin–clavulanic acid at clinically relevant doses using the in vitro Simulator of the Human Intestinal Microbial Ecosystem (SHIME^®^). Furthermore, the impact of prolonged repeated dosing of lactulose following antibiotic treatment was investigated in terms of microbiome recovery.

## 2. Results

### 2.1. Analysis of the Microbial Metabolic Activity

**Figure 1:**
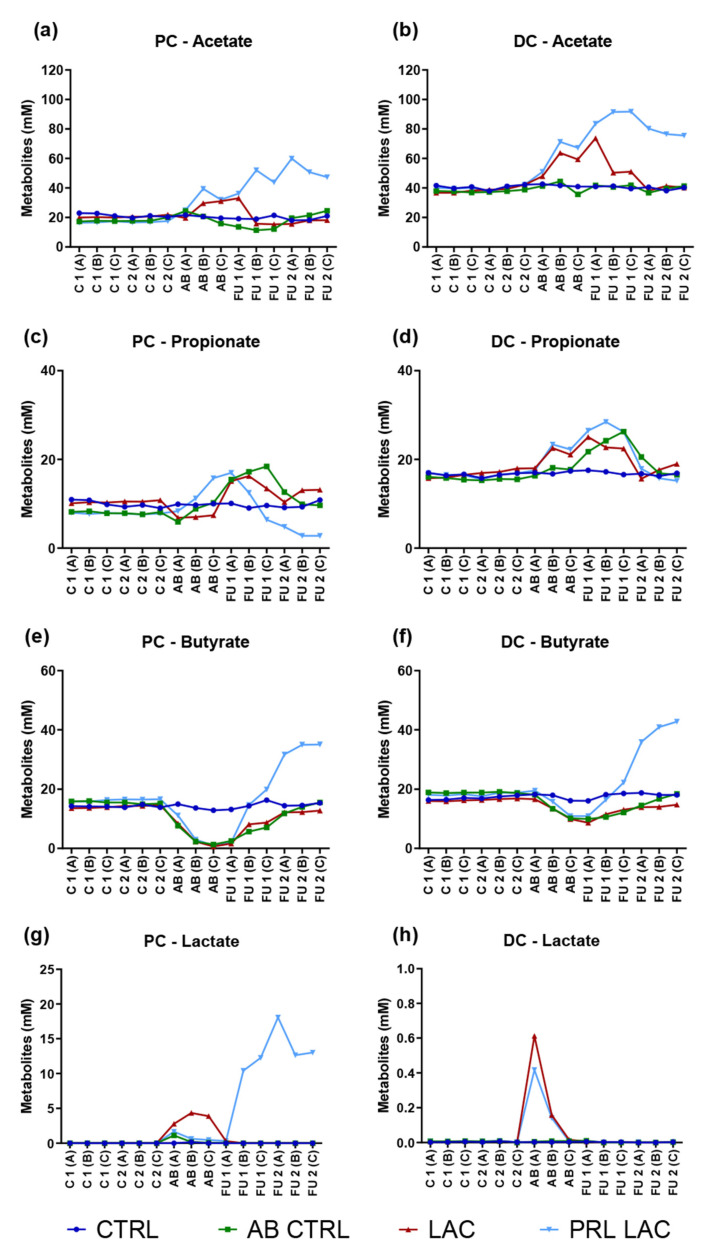
Microbial metabolic activity over time. Absolute concentrations (mM) of acetate (**a**,**b**), propionate (**c**,**d**), butyrate (**e**,**f**) and lactate (**g**,**h**) in the proximal (PC) and distal colon (DC) reactors upon lactulose administration at a dose of 5 g/d during antibiotic treatment (LAC) as well as lactulose treatment during and prolonged following antibiotic treatment (PRL LAC) compared to an antibiotic control (AB CTRL) and a negative control (CTRL). Samples were taken during two control (C1 and C2), one antibiotic (AB) and two follow-up (FU1 and FU2) weeks. During each week, three samples were collected; these are shown separately in the graph.

Short-chain fatty acid (SCFA) levels were stable during the control period within the different SHIME arms. Similarly, the negative control incubations (CTRL) were characterized by stable levels during the entire experimental period (Figure 1 and Supplementary Figure S1).

Antibiotic treatment in the absence of lactulose supplementation (AB CTRL) resulted in a slight decrease in acetate levels in both colon regions (Figure 1 and Figure S1), though not reaching statistical significance. Butyrate levels were more strongly affected, resulting in a significant reduction (*p* < 0.001 in the proximal colon (PC)) of butyrate levels at the end of the antibiotic treatment period (Table 1 and Table S1). On the other hand, propionate production was stimulated towards the end of the antibiotic treatment in both colon regions, reaching significance in the distal colon (DC). Upon cessation of antibiotic treatment, propionate levels increased even further in the absence of lactulose supplementation.

Addition of lactulose during the antibiotic treatment resulted in significantly enhanced acetate levels as compared to the control period, i.e., an average increase of 10.7 mM (or +57% as compared to the control period) and 20.7 mM (+53%) upon supplementation of 5 g lactulose per day and an average increase of 14.2 mM (+74%) and 31.0 mM (+78%) upon administration of 10 g/d lactulose in the PC and DC, respectively (Table 1 and Table S1). While cessation of lactulose treatment resulted in a reduction in acetate levels, prolonged lactulose administration further increased acetate concentrations, reaching significantly higher levels as compared to the other test conditions at the end of the follow-up period in both PC (*p* < 0.001 and *p* = 0.008 for the 5 g/d and 10 g/d test dose, respectively) and DC (*p* < 0.001 and *p* = 0.003 for the 5 g/d and 10 g/d test dose, respectively). Overall, final acetate levels were similar for both doses of lactulose tested, though during antibiotic treatment a trend towards increased acetate levels, especially in the DC, was observed when administering the higher dose of lactulose (on average, acetate levels were 31.6 and 34.6 mM in PC and 63.3 and 78.5 mM in DC upon dosing 5 and 10 g/d of lactulose, respectively). While the addition of lactulose during the antibiotic treatment resulted in a similar stimulation of propionate levels as observed for the antibiotic control, prolonged lactulose administration (at both doses tested) significantly reduced propionate levels during the follow-up period in the PC, resulting in an average decrease of 8.3 mM (−71%) and 0.6 mM (−10%) at 5 g/d and 10 g/d, respectively, when compared to the antibiotic treatment period.

In terms of butyrate production, cessation of antibiotic treatment resulted in recovery of butyrate levels, with the final butyrate concentrations reaching similar levels to those observed during the control period in the absence of lactulose supplementation. The addition of lactulose during the antibiotic period resulted in similar trends, though administration of the higher lactulose dose during the antibiotic period showed faster recovery of butyrate levels. Prolonged administration of lactulose during follow-up, on the other hand, increased butyrate levels beyond concentrations observed during the control period, resulting in significantly higher levels at the end of the experiment in both colon regions, i.e., an average increase of 17.7 mM (or +109% as compared to the control period) and 21.6 mM (+118%) upon supplementation of 5 g lactulose per day and an average increase of 16.7 mM (+107%) and 47.3 mM (+262%) upon administration of 10 g/d lactulose in the PC and DC, respectively.

Finally, the addition of lactulose resulted in a strong stimulation of lactate levels, followed by a reduction in lactate levels upon cessation of lactulose treatment. Prolonged lactulose administration resulted in a further increase in lactate levels in the PC during the follow-up period, with the strongest increases observed when supplementing the higher dose of lactulose (i.e., an average increase of 13.0 mM as compared to the control period).

With respect to markers for proteolytic fermentation (Table 2 and Table S2), antibiotic treatment reduced ammonium and branched-chain fatty acid (BCFA) levels in both colon regions. After cessation of antibiotic and lactulose treatment, recovery of ammonium and BCFA levels was observed in both colon regions. Prolonged lactulose supplementation during follow-up, on the other hand, limited ammonium and BCFA recovery, resulting in significantly lower levels as compared to the control period (*p* < 0.001).

### 2.2. Analysis of Microbial Community Composition

**Figure 2:**
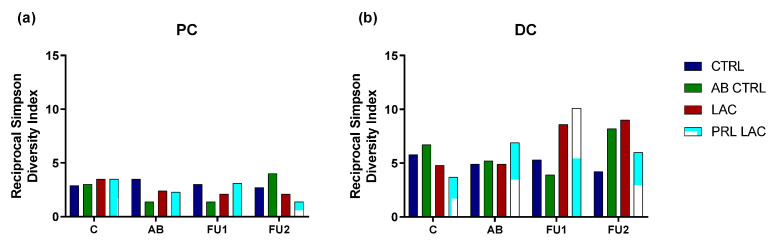
Reciprocal Simpson Diversity Index. Reciprocal Simpson diversity index in the proximal (PC (**a**)) and distal colon (DC (**b**)) reactors at the end of the control period (C), antibiotic treatment (AB) and follow-up weeks 1 (FU1) and 2 (FU2) upon lactulose administration at a dose of 5 g/d during antibiotic treatment (LAC) as well as lactulose administration during and prolonged following antibiotic treatment (PRL LAC) compared to an antibiotic control (AB CTRL) and a negative control (CTRL) (n = 1).

The reciprocal Simpson diversity index was calculated as a measure of diversity (Figure 2 and Figure S2). Antibiotic treatment decreased microbial diversity in the PC. Administration of lactulose tended to protect the diversity of the microbial community, as seen by lower reductions in the reciprocal Simpson diversity index as compared to the antibiotic control. Upon cessation of antibiotic treatment, an increase in microbial diversity was observed towards the end of the experimental period. However, prolonged lactulose supplementation resulted in a reduction in microbial diversity towards the end of the follow-up period in both colon regions.

**Figure 3:**
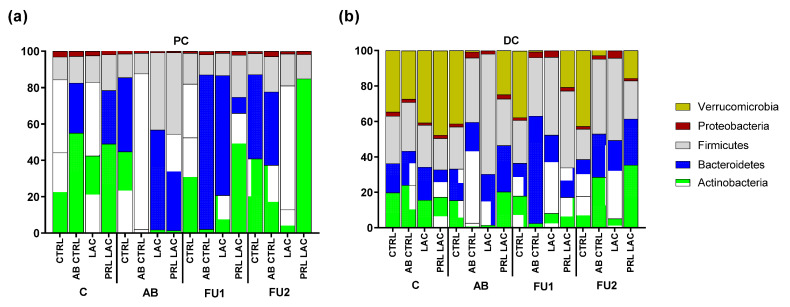
Microbial community composition as assessed via 16S-targeted Illumina sequencing. Abundance (%) at microbial phylum level in the proximal (PC (**a**)) and distal colon (DC (**b**)) reactors at the end of the control period (C), antibiotic treatment (AB) and follow-up weeks 1 (FU1) and 2 (FU2) upon lactulose administration at a dose of 5 g/d during antibiotic treatment (LAC) as well as lactulose administration during and prolonged following antibiotic treatment (PRL LAC) compared to an antibiotic control (AB CTRL) and a negative control (CTRL) (n = 1).

With respect to microbial community composition (Figure 3 and Figure S3, Table 3 and Table 4, Tables S3 and S4), the antibiotic treatment resulted in a reduction in Actinobacteria levels in both colon regions, as well as strong decreases in *Akkermansiaceae* in the DC. The decrease in Actinobacteria levels seen after antibiotic application was mainly linked to large decreases in *Bifidobacteriaceae* abundance, which was compensated by increased abundance of *Bacteroidaceae*. Furthermore, antibiotic treatment specifically resulted in enrichment of Proteobacteria, as evidenced by increased abundances of several families belonging to the Proteobacteria phylum (except in the PC of the QuadSHIME that was dosed with 5 g lactulose per day). Dosing lactulose during the antibiotic treatment period resulted in increased levels of Firmicutes at the expense of Bacteroidetes. The increased Firmicutes levels were attributed to increased abundance of *Lachnospiraceae* and *Lactobacillaceae*. Co-administration of lactulose resulted in a strong recovery of *Bifidobacteriaceae* levels following antibiotic treatment (especially in the PC) as compared to the control, with an additional strong stimulatory effect being observed for the prolonged lactulose application.

The strong stimulation of *Bifidobacteriaceae* with the extended lactulose supplementation was accompanied by a decrease in *Bacteroidaceae* and *Lachnospiraceae*. Several other bacterial groups were also stimulated when lactulose was given during the follow-up period, including *Rikenellaceae* in the DC and *Veillonellaceae* in both colon regions. *Tannerellaceae* levels also increased in the DC, though only upon prolonged administration of lactulose at a dose of 5 g/d. Furthermore, following antibiotic treatment, increased abundance of *Enterobacteriaceae* was observed in the PC in the absence of lactulose treatment (observed to a larger extent in the QuadSHIME that was dosed with 10 g lactulose per day), while lactulose application limited this effect, especially when supplementation was continued.

## 3. Discussion

The main finding of the present study is that metabolite production was stable and reproducible within and between the different SHIME units, while the negative control incubation was also characterized by stable levels during the entire experimental period (5 weeks). These observations are in line with previous findings of Possemiers et al. which demonstrated that a stable microbial community was reached within 2 weeks after the inoculation of fecal material into reactors [21]. Furthermore, our results are supported by the findings of Van den Abbeele et al. which showed that reproducible microbial communities were obtained in independent reactors when using the same fecal inoculum, allowing a direct comparison between the test conditions for virtually identical microbial communities [22].

In addition, our study demonstrated that amoxicillin–clavulanic acid treatment at clinically relevant concentrations strongly decreased *Bifidobacteriaceae* levels in both PC and DC, thereby confirming previous in vivo studies, including antibiotic therapy [7,9]. Furthermore, a strong decrease in acetate and mostly butyrate levels was observed. This is in accordance with earlier findings of Ladirat et al. which demonstrated that amoxicillin treatment resulted in a drastic reduction in butyrate production in vitro [8]. Similarly, several in vivo studies have already reported decreased abundance of the butyrate-producing *Lachnospiraceae* and *Ruminococcaceae* families following amoxicillin treatment [7,8,23]. In addition to the reduction in numbers of butyrate-producing micro-organisms, the propionate-producing *Bacteroidaceae* family was strongly affected by amoxicillin therapy in these former studies [8,23], though conflicting results have been reported [24,25].

In the current study, propionate production was stimulated towards the end of the antibiotic treatment, which correlated with increased relative abundance of *Bacteroidaceae*. *Bacteroidaceae* could potentially obtain a competitive advantage upon partial eradication of *Bifidobacterium* and butyrate-producing species with amoxicillin–clavulanic acid treatment.

During the antibiotic treatment, the co-administration of lactulose resulted in significantly enhanced levels of acetate and lactate in both colon regions, which was associated with increased abundance of *Lactobacillaceae*. Similarly, studies performed by Bothe et al. [16,18] showed that daily administration of lactulose specifically stimulated the growth of *Lactobacillus* species under healthy conditions in vitro, while Nikolaou et al. [20] reported increased abundance of lactobacilli upon lactulose supplementation following azithromycin treatment in vivo. Overall, these results indicate the fermentation of lactulose by *Lactobacillus* spp. even during antibiotic treatment.

Following amoxicillin–clavulanic acid treatment, co-administration of lactulose during antibiotic therapy resulted in a marked recovery of *Bifidobacteriaceae* as compared to the antibiotic control (especially in the PC), with an additional stimulatory effect being observed when the application of lactulose was continued. Furthermore, it was observed that, post-treatment with amoxicillin–clavulanic acid, butyrate levels recovered in both colon regions, with the strongest recovery upon prolonged administration of lactulose. The butyrogenic effect was optimal towards the end of the administration period, probably because of the establishment of cross-feeding interactions between *Bifidobacteriaceae* and butyrate-producing bacteria [26,27].

At the community level, next to enhanced *Bifidobacteriaceae* abundance, the expanded supplementation with lactulose was accompanied by strongly reduced Bacteroidetes levels, while the higher dose of lactulose significantly increased Firmicutes levels towards the end of the follow-up period. This finding is in line with the observations of Ladirat et al. [7] which showed that prebiotic intake stimulated recovery of *Bifidobacterium* species as well as butyrate-producing bacteria belonging to the Firmicutes phylum following amoxicillin treatment. Moreover, extended lactulose administration in our study resulted in increased abundance of *Rikenellaceae* in the DC, a finding that is supported by Duysburgh and co-workers [19]. Overall, these results indicate that administration of lactulose might be effective in restoring the gut microbiome following amoxicillin–clavulanic acid treatment by promoting *Lactobacillus* and *Bifidobacterium* species, which in turn could stimulate cross-feeding interactions.

With respect to markers for proteolytic fermentation, antibiotic treatment reduced ammonium and BCFA levels in both colon regions. Following antibiotic treatment, recovery of ammonium and BCFA levels was observed in both colon regions, which was accompanied with an outgrowth of *Enterobacteriaceae* in the PC. Lactulose supplementation during and after antibiotic treatment, on the other hand, limited ammonium and BCFA recovery, thereby controlling *Enterobacteriaceae* outgrowth. While the *Enterobacteriaceae* family contains many commensal gut microbiota, this family is mostly linked with enteric diseases as it also contains several pathogenic micro-organisms, such as *Escherichia coli*, *Salmonella*, *Enterobacter* and *Shigella* [28], indicating that lactulose supplementation might inhibit the colonization of opportunistic pathogenic species following antibiotic therapy. In this context, Nikolaou et al. [20] reported that the relative abundance of opportunistic pathogens (e.g., *Streptococcus*) significantly increased following treatment with the antibiotic azithromycin in children, while co-administration of lactulose with azithromycin limited the outgrowth of these opportunistic pathogens. This suggests that lactulose supplementation promotes recovery of the gut microbiome following antibiotic therapy, thereby providing a protective role against pathogenic colonization.

This study has some limitations that we would like to acknowledge. First, inter-individual variations were not evaluated. However, it is important to note that in vitro gut models depend on the microbiome present in human stool samples and that human microbial community composition is generally characterized by large inter-individual differences [29]. Therefore, a follow-up study could be envisioned in which more donors are included to investigate the potential inter-individual response. Second, host–microbiome interactions were not investigated during the current study. Combining samples obtained from the SHIME^®^ model with a co-culture human cell model [30] could have provided insight into the immune-modulatory properties of lactulose following antibiotic therapy but this was outside the scope of our experiment. Lastly, the potential gastrointestinal side effects of consuming lactulose at high doses were not investigated in the current study. High dosages of lactulose (i.e., 10 g/day or higher) are typically provided to alleviate constipation [12], and therefore consumption of high doses lactulose by non-constipated individuals might result in gastrointestinal side effects, such as diarrhoea. Even though in the current study a lower lactulose dose (i.e., 5 g/day) was included to investigate whether this lower dosage still had the potential to support the microbiome accordingly, while no to limited side effects were expected, this should still be further examined in in vivo trials. Taken together, the results show that amoxicillin–clavulanic acid treatment resulted in a strong decrease in acetate and butyrate levels, indicating the formation of a dysbiosed microbial community. During the antibiotic period, administration of lactulose resulted in strongly enhanced acetate and lactate levels in the PC and DC, probably due to the fermentation of lactulose by primary substrate degraders, such as *Bifidobacterium* and *Lactobacillus* species. This subsequently stimulated the recovery of butyrate, with a strong butyrogenic effect being observed upon prolonged lactulose supplementation after cessation of antibiotic administration, with the strongest effects being observed for the higher dose of lactulose (10 g) tested. The current study supports the view that lactulose is an interesting candidate in limiting the detrimental effects of amoxicillin–clavulanic acid on the human gut microbiome. Clinical studies are now warranted to confirm these promising findings.

## 4. Materials and Methods

### 4.1. Chemicals and Test Product

All chemicals were obtained from Sigma-Aldrich (Overijse, Belgium) unless stated otherwise. Fresenius-Kabi iPSUM S.r.l. (Vicchio, Italy) provided the lactulose crystals (Ph.Eur.), which were tested at two different dosages, i.e., an in vitro dose of 5 g per day and an in vitro dose of 10 g per day. While the highest dosage (i.e., 10 g/day) corresponds to the (lowest) approved dose for medicinal application (typically to alleviate constipation), the lower dosage (i.e., 5 g/day) was selected to investigate whether this dose would still have the potential to support the microbiome while limiting the risk of gastrointestinal side effects (e.g., diarrhoea).

### 4.2. Simulator of the Human Intestinal Microbial Ecosystem (SHIME^®^)

In vitro simulation of the human gastrointestinal tract was derived from the SHIME^®^ model (ProDigest and Ghent University, Gent, Belgium) as described by Molly et al. [31]. The reactor configuration was adapted from a single SHIME setup (including 1 SHIME arm) to a QuadSHIME setup (including 4 SHIME arms) in order to allow the study of four different test conditions in one single setup [32]. Each arm of the QuadSHIME consisted of a succession of three reactors simulating the different parts of the gastrointestinal tract. The first reactor mimicked the upper gastrointestinal tract, including subsequent simulation of the stomach and small intestine. The two colonic reactors simulated the PC, operated at pH 5.6–5.9 with a retention time of 20 h, and the DC, operated at pH 6.6–6.9 with a retention time of 32 h. The inoculum preparation, temperature settings, feeding regime and reactor feed composition were adopted from Possemiers et al. [21]. Upon inoculation with a fecal sample from a healthy human adult donor (female, 24 y), a two-week stabilization period was initiated to allow the microbial community to differentiate in the different colonic reactors depending on the local environmental conditions. Subsequently, the baseline microbial community composition and activity were determined in the different reactors during a two-week control period. Next, antibiotic treatment was initiated. Amoxicillin–clavulanic acid (2:1; Toku-E, Sint-Denijs-Westrem, Belgium) was dosed to the PC reactors of each arm at a concentration of 150 mg/day (50 mg/feeding cycle) for a period of 5 days. This daily dosage corresponds to the maximum in vivo amount that would reach the colon. Indeed, in vivo daily doses of 1500 mg amoxicillin per day are prescribed, generally divided over three administrations per day [33]. Out of this total oral dose, 90% is absorbed in the small intestine, meaning that not more than 10% would enter the colon [34].

Amoxicillin is generally administered together with clavulanic acid in a clavulanic acid:amoxicillin ratio of 1:4 or 1:7 in vivo. Out of this oral dose of clavulanic acid, around 73% is absorbed in vivo [35]. If the original clavulanic acid:amoxicillin ratio in the product is 1:4 or 1:7, then after absorption at a rate of 73%:90%, this would result in a ratio of 1:1.5 up to 1:2.6. Therefore, a clavulanic acid:amoxicillin ratio of 1:2 was used in this study. During the 5-day antibiotic period, the four arms of the QuadSHIME were operated as follows: (1) control arm (CTRL), not receiving antibiotic treatment; (2) only antibiotic treatment (AB CTRL); (3) antibiotic treatment together with supplementation of lactulose once per day (LAC); and (4) antibiotic treatment together with supplementation of lactulose once per day (LAC PRL). After cessation of antibiotic treatment, a follow-up period of two weeks was initiated in which the QuadSHIME was operated as during the control period to assess the recovery of the gut microbiota in terms of composition and activity. In arm (4) of the QuadSHIME (LAC PRL), lactulose was supplemented once per day on top of the standard nutritional matrix during this follow-up period. Two QuadSHIME systems were operated in parallel to assess the efficacy of two different doses of lactulose, i.e., 5 and 10 g per day.

### 4.3. Microbial Metabolic Activity

Starting from the control period, samples for analysis of microbial metabolic activity were collected three times per week from each colonic vessel. SCFA levels were determined as described by De Weirdt et al. [36] and included measurement of acetate, propionate, butyrate and BCFA (including isobutyrate, isovalerate and isocaproate) concentrations. Lactic acid determination was conducted using a commercially available enzymatic assay kit (R-Biopharm, Darmstadt, Germany) according to the manufacturer’s instructions. Ammonium analysis was performed as previously described by Duysburgh et al. [37].

### 4.4. Microbial Community Analysis

Starting from the final week during the control phase, samples for microbiota profiling were collected once per week from each colonic vessel. DNA was isolated as previously described by Boon et al. [38], using pelleted cells originating from a 1 mL luminal sample. Subsequently, the assessment of microbial community composition of each colon compartment was established by 16S-targeted Illumina sequencing analysis. Using primers 341F (5′-CCT ACG GGN GGC WGC AG-3′) and 785Rmod (5′-GAC TAC HVG GGT ATC TAA KCC-3′), adapted from Klindworth et al. [39], the 16S rRNA gene V3–V4 hypervariable regions were amplified by PCR. The original genomic DNA extracts were diluted in DNase/RNase/protease-free water to obtain a concentration of 50 ng/µL and 30 µL was send out to LGC genomics GmbH (Germany) for library preparation and sequencing on an Illumina Miseq platform with v3 chemistry with the primers mentioned above.

### 4.5. Statistics

The comparison of normally distributed data for the different experimental periods on microbial metabolic markers was performed with a Student’s *t*-test for pairwise comparisons. The normality of the dataset was confirmed based on historical data. Differences were considered significant if *p* < 0.05. Data are presented as means ± standard deviations. Statistically significant differences relative to the control period are indicated in bold.

For the 16S-targeted Illumina sequencing analysis, read assembly and clean-up was largely derived from the MiSeq procedure as previously described [40,41]. In brief, mothur (v. 1.42.0) was used to assemble reads into contigs, perform alignment-based quality filtering (alignment to the mothur-reconstructed SILVA SEED alignment, v. 123), remove chimeras (vsearch, v. 2.13.0), assign taxonomy using a naïve Bayesian classifier [42] and SILVA NR (v. 132), and cluster contigs into Operational Taxonomic Units (OTUs) at 97% sequence similarity. Sequences classified as Eukaryota, Archaea, Chloroplasts and Mitochondria and sequences that could not be classified at all (even at (super-)kingdom level) were removed. For each OTU, representative sequences were picked as the most abundant sequence within that OTU.

## Figures and Tables

**Table 1:** Overall metabolic activity in terms of SCFA and lactic acid production. Average acetate, propionate, butyrate and lactate production (mM) during the control (C; n = 6), the antibiotic (AB; n = 3) and the two follow-up (FU1/2; n = 3) weeks in the proximal (PC) and distal colon (DC) reactors upon lactulose administration at a dose of 5 g/d during antibiotic treatment (LAC) as well as lactulose co-administration during and prolonged following antibiotic treatment (PRL LAC) compared to an antibiotic control (AB CTRL) and a negative control (CTRL). Data are presented as means ± SDs. Statistically significant differences relative to the control period are indicated in bold (*p* < 0.05). The intensity of shading indicates the absolute concentration, normalized for each of the different metabolites per colonic region. Lowest values are indicated with two shades of red, medium values are indicated in white, whereas the highest values are indicated with two shades of green.

			C		AB		FU1		FU2	
Acetate (mM)	PC	CTRL	21.3	(±1.3)	20.6	(±1.0)	19.8	(±1.4)	19.1	(±1.6)
		AB CTRL	18.0	(±1.0)	20.3	(±4.3)	**12.4**	(±1.2)	**21.8**	(±2.5)
		LAC	20.5	(±0.7)	**26.8**	(±6.2)	21.4	(±10.1)	**17.3**	(±1.4)
		PRL LAC	16.9	(±0.4)	**32.2**	(±7.3)	**44.0**	(±7.9)	**52.7**	(±6.5)
	DC	CTRL	40.6	(±1.5)	41.7	(±0.9)	40.6	(±0.9)	39.6	(±1.3)
		AB CTRL	37.8	(±0.7)	40.5	(±4.5)	**41.4**	(±0.7)	39.2	(±2.3)
		LAC	38.7	(±2.0)	**57.0**	(±8.1)	**58.4**	(±13.3)	39.9	(±1.6)
		PRL LAC	40.0	(±1.5)	**63.1**	(±10.7)	**89.0**	(±4.7)	**77.4**	(±2.4)
Propionate (mM)	PC	CTRL	9.9	(±0.8)	9.9	(±0.2)	9.6	(±0.5)	9.8	(±0.9)
		AB CTRL	8.0	(±0.3)	8.3	(±2.2)	**17.0**	(±1.5)	**10.7**	(±1.7)
		LAC	10.4	(±0.2)	**7.1**	(±0.3)	**14.9**	(±1.4)	**12.2**	(±1.6)
		PRL LAC	7.8	(±0.1)	**11.8**	(±3.7)	12.0	(±5.3)	**3.5**	(±1.2)
	DC	CTRL	16.5	(±0.4)	17.1	(±0.3)	17.1	(±0.5)	16.6	(±0.3)
		AB CTRL	15.6	(±0.3)	**17.4**	(±0.9)	**24.1**	(±2.3)	**18.0**	(±2.2)
		LAC	16.7	(±0.8)	**20.6**	(±2.3)	**23.4**	(±1.4)	17.4	(±1.7)
		PRL LAC	16.5	(±0.5)	**21.1**	(±3.1)	**27.0**	(±1.2)	16.3	(±1.4)
Butyrate (mM)	PC	CTRL	14.2	(±0.3)	13.8	(±1.0)	14.6	(±1.6)	14.8	(±0.5)
		AB CTRL	15.5	(±0.4)	**3.8**	(±3.4)	**5.1**	(±2.4)	13.8	(±1.8)
		LAC	14.1	(±0.4)	**3.8**	(±4.1)	**6.1**	(±4.0)	**12.4**	(±0.3)
		PRL LAC	16.3	(±0.4)	**5.0**	(±5.4)	12.2	(±9.2)	**34.0**	(±1.9)
	DC	CTRL	17.0	(±0.6)	17.5	(±1.2)	17.6	(±1.3)	**18.3**	(±0.4)
		AB CTRL	18.9	(±0.1)	**13.9**	(±4.0)	**10.9**	(±1.1)	**16.6**	(±1.9)
		LAC	16.4	(±0.4)	13.3	(±3.3)	**11.1**	(±2.2)	**14.3**	(±0.5)
		PRL LAC	18.3	(±0.5)	15.4	(±4.3)	16.5	(±5.7)	**39.9**	(±3.5)
Lactate (mM)	PC	CTRL	0.01	(±0.01)	0.05	(±0.09)	0.01	(±0.00)	**0.00**	(±0.00)
		AB CTRL	0.01	(±0.00)	0.46	(±0.57)	**0.01**	(±0.00)	**0.00**	(±0.00)
		LAC	0.01	(±0.00)	**3.67**	(±0.81)	0.11	(±0.17)	**0.00**	(±0.00)
		PRL LAC	0.01	(±0.00)	**0.92**	(±0.64)	**7.67**	(±6.43)	**14.58**	(±3.04)
	DC	CTRL	0.004	(±0.000)	0.004	(±0.000)	0.003	(±0.000)	0.001	(±0.000)
		AB CTRL	0.006	(±0.000)	0.008	(±0.000)	0.003	(±0.010)	**0.001**	(±0.000)
		LAC	0.006	(±0.000)	0.262	(±0.310)	0.003	(±0.000)	**0.001**	(±0.000)
		PRL LAC	0.007	(±0.000)	0.189	(±0.210)	0.003	(±0.010)	**0.002**	(±0.000)

**Table 2:** Microbial metabolic activity in terms of BCFA and ammonium production. Average BCFA (mM) and ammonium (mg/L) production during the control (C; n = 6), the antibiotic (AB; n = 3) and the two follow-up (FU1/2; n = 3) weeks in the proximal (PC) and distal colon (DC) reactors upon lactulose administration at a dose of 5 g/d during antibiotic treatment (LAC) as well as lactulose administration during and prolonged following antibiotic treatment (PRL LAC) compared to an antibiotic control (AB CTRL) and a negative control (CTRL). Data are presented as means ± SDs. Statistically significant differences relative to the control period are indicated in bold (*p* < 0.05). The intensity of shading indicates the absolute concentration, normalized for each of the different metabolites per colonic region. Lowest values are indicated with two shades of red, medium values are indicated in white, whereas the highest values are indicated with two shades of green.

			C		AB		FU1		FU2	
BCFA (mM)	PC	CTRL	2.82	(±0.11)	2.66	(±0.24)	**2.61**	(±0.10)	**2.59**	(±0.19)
		AB CTRL	2.80	(±0.05)	**0.92**	(±0.58)	**2.22**	(±0.57)	**2.64**	(±0.09)
		LAC	2.76	(±0.04)	**0.87**	(±1.15)	1.80	(±1.26)	2.66	(±0.13)
		PRL LAC	2.66	(±0.05)	**0.85**	(±1.00)	**1.74**	(±0.80)	**1.79**	(±0.07)
	DC	CTRL	2.95	(±0.05)	2.92	(±0.28)	2.86	(±0.21)	2.92	(±0.03)
		AB CTRL	3.01	(±0.09)	**2.62**	(±0.40)	**2.12**	(±0.26)	2.88	(±0.13)
		LAC	2.88	(±0.11)	**1.70**	(±1.01)	**1.66**	(±1.13)	2.73	(±0.15)
		PRL LAC	2.93	(±0.10)	**1.62**	(±1.10)	**1.30**	(±0.26)	**2.15**	(±0.02)
Ammonium (mg/L)	PC	CTRL	232	(±7)	225	(±29)	231	(±22)	220	(±25)
		AB CTRL	210	(±12)	**143**	(±38)	203	(±35)	**238**	(±1)
		LAC	232	(±10)	**139**	(±50)	**182**	(±44)	243	(±2)
		PRL LAC	203	(±10)	**138**	(±36)	**150**	(±25)	**98**	(±16)
	DC	CTRL	351	(±13)	345	(±14)	365	(±22)	353	(±29)
		AB CTRL	359	(±17)	345	(±20)	334	(±65)	362	(±6)
		LAC	339	(±17)	**236**	(±102)	274	(±74)	309	(±53)
		PRL LAC	348	(±11)	**240**	(±93)	**223**	(±27)	**233**	(±27)

**Table 3:** Proximal microbial community composition as assessed via 16S-targeted Illumina sequencing at family level. Abundance (%) at microbial family level in the proximal colon (PC) reactor at the end of the control period (C), antibiotic treatment (AB) and follow-up weeks 1 (FU1) and 2 (FU2) upon lactulose administration at a dose of 5 g/d during antibiotic treatment (LAC) as well as lactulose administration during and prolonged following antibiotic treatment (PRL LAC) compared to an antibiotic control (AB CTRL) and a negative control (CTRL) (n = 1). The intensity of shading indicates the absolute abundance, normalized for each of the different families. Lowest values are indicated with two shades of red, medium values are indicated in white, whereas the highest values are indicated with two shades of green.

Phylum	Family	PC															
		C				AB				FU1				FU2			
		CTRL	AB CTRL	LAC	PRL LAC	CTRL	AB CTRL	LAC	PRL LAC	CTRL	AB CTRL	LAC	PRL LAC	CTRL	AB CTRL	LAC	PRL LAC
Actinobacteria	*Bifidobacteriaceae*	44.2	54.4	42.3	48.6	44.5	0.6	1.3	0.5	52.4	1.0	20.4	64.8	40.8	37.0	12.8	84.7
	*Microbacteriaceae*	0.0	0.4	0.1	0.4	0.1	1.2	0.5	0.8	0.0	1.1	0.2	1.0	0.0	0.2	0.1	0.1
Bacteroidetes	*Bacteroidaceae*	40.1	27.5	40.6	29.4	40.8	85.6	54.9	53.0	29.5	84.9	65.9	8.9	46.3	40.3	68.1	0.0
Firmicutes	*Acidaminococcaceae*	1.2	2.6	1.2	1.7	2.0	0.4	0.0	2.5	1.5	3.6	1.9	0.8	1.5	1.9	2.7	0.2
	*Enterococcaceae*	0.2	0.5	0.3	0.0	0.5	1.5	0.5	0.0	0.7	1.0	0.7	0.1	0.2	0.5	0.7	0.5
	*Lachnospiraceae*	9.0	3.6	11.8	10.7	8.2	6.5	31.2	40.2	12.1	5.8	7.9	9.9	9.1	11.3	12.4	9.0
	*Lactobacillaceae*	1.3	3.6	0.3	2.6	0.3	2.8	10.7	2.1	0.4	0.3	1.1	2.9	0.1	0.1	0.3	0.1
	*Ruminococcaceae*	0.0	0.0	0.0	0.0	0.0	0.0	0.0	0.0	0.0	0.0	0.0	0.1	0.0	0.0	0.0	0.0
	*Veillonellaceae*	0.8	4.3	1.1	4.7	1.9	0.1	0.0	0.0	2.2	0.4	0.7	9.1	0.8	5.7	1.3	3.4
Proteobacteria	*Burkholderiaceae*	0.4	0.6	0.6	0.6	0.4	0.0	0.1	0.1	0.5	0.2	0.4	0.7	0.3	1.0	0.2	1.2
	*Desulfovibrionaceae*	2.5	1.6	1.1	0.3	0.8	0.1	0.1	0.0	0.5	0.8	0.2	0.0	0.4	0.9	0.8	0.0
	*Enterobacteriaceae*	0.1	0.3	0.6	0.3	0.1	0.0	0.3	0.1	0.0	0.5	0.2	0.2	0.0	0.2	0.2	0.1
	*Pseudomonadaceae*	0.1	0.3	0.0	0.7	0.3	0.9	0.3	0.5	0.1	0.2	0.2	1.1	0.4	0.7	0.2	0.4
	*Xanthomonadaceae*	0.0	0.1	0.0	0.0	0.0	0.1	0.0	0.0	0.0	0.1	0.1	0.0	0.0	0.1	0.0	0.0

**Table 4:** Distal microbial community composition as assessed via 16S-targeted Illumina sequencing at family level. Abundance (%) at microbial family level in the distal colon (DC) reactor at the end of the control period (C), antibiotic treatment (AB) and follow-up weeks 1 (FU1) and 2 (FU2) upon lactulose administration at a dose of 5 g/d during antibiotic treatment (LAC) as well as lactulose administration during and prolonged following antibiotic treatment (PRL LAC) compared to an antibiotic control (AB CTRL) and a negative control (CTRL) (n = 1). The intensity of shading indicates the absolute abundance, normalized for each of the different families. Lowest values are indicated with two shades of red, medium values are indicated in white, whereas the highest values are indicated with two shades of green.

Phylum	Family	DC															
		C				AB				FU1				FU2			
		CTRL	AB CTRL	LAC	PRL LAC	CTRL	AB CTRL	LAC	PRL LAC	CTRL	AB CTRL	LAC	PRL LAC	CTRL	AB CTRL	LAC	PRL LAC
Actinobacteria	*Bifidobacteriaceae*	19.6	23.8	15.5	17.2	15.3	2.3	1.4	20.0	17.9	2.2	7.6	16.9	17.7	28.4	5.0	34.9
	*Coriobacteriaceae*	0.0	0.1	0.0	0.0	0.0	0.0	0.0	0.0	0.0	0.0	0.0	0.0	0.0	0.0	0.0	0.1
	*Microbacteriaceae*	0.0	0.0	0.0	0.0	0.0	0.3	0.1	0.1	0.0	0.2	0.5	0.2	0.0	0.1	0.1	0.3
Bacteroidetes	*Bacteroidaceae*	10.9	9.3	11.4	7.8	13.0	50.4	23.2	22.3	14.1	59.5	41.9	9.7	18.7	19.8	35.8	18.5
	*Prevotellaceae*	1.0	2.4	2.0	1.8	1.1	4.0	0.3	2.1	2.0	0.1	0.0	0.2	0.7	0.6	5.1	1.5
	*Rikenellaceae*	0.1	0.0	0.0	0.0	0.1	0.0	0.1	0.1	0.0	0.0	0.1	1.5	0.0	0.1	0.0	2.1
	*Tannerellaceae*	4.5	7.6	5.2	6.0	3.5	2.3	5.3	1.6	2.4	1.0	2.2	5.2	1.3	3.9	3.3	4.0
Firmicutes	*Acidaminococcaceae*	0.7	0.6	1.0	0.5	0.9	0.6	1.3	0.4	1.1	1.1	2.3	0.9	1.3	1.2	1.9	0.3
	*Enterococcaceae*	0.0	0.0	0.1	0.0	0.0	0.1	0.1	0.0	0.1	0.1	0.1	0.0	0.0	0.1	0.1	0.0
	*Lachnospiraceae*	22.9	23.5	21.3	16.1	20.4	25.1	59.9	24.5	21.0	23.6	32.5	40.2	14.1	34.8	42.9	20.1
	*Lactobacillaceae*	0.2	0.1	0.0	0.1	0.1	0.7	2.7	0.5	0.1	0.1	0.8	0.4	0.0	0.1	0.1	0.0
	*Ruminococcaceae*	2.9	3.2	1.2	0.8	2.0	9.3	4.0	0.7	1.8	7.5	7.2	1.5	1.6	5.8	1.0	0.2
	*Veillonellaceae*	0.1	0.1	0.1	0.1	0.3	0.5	0.0	0.1	0.1	0.6	1.1	0.3	0.1	0.2	0.4	0.9
Proteobacteria	*Burkholderiaceae*	0.2	0.3	0.1	0.2	0.2	1.1	0.6	1.0	0.1	0.4	0.5	0.7	0.2	0.4	0.3	0.3
	*Desulfovibrionaceae*	1.8	1.3	1.1	1.3	1.2	1.3	0.7	0.5	1.0	0.7	2.5	0.8	0.7	0.8	1.3	0.4
	*Enterobacteriaceae*	0.0	0.0	0.1	0.0	0.0	0.0	0.0	0.0	0.0	0.0	0.0	0.0	0.0	0.0	0.0	0.0
	*Pseudomonadaceae*	0.1	0.1	0.1	0.1	0.2	0.2	0.1	0.1	0.3	0.3	0.2	0.2	0.2	0.7	0.2	0.1
	*uncultured*	0.2	0.2	0.1	0.2	0.1	0.6	0.3	0.9	0.1	1.8	0.3	0.3	0.4	0.0	2.3	0.5
Verrucomicrobia	*Akkermansiaceae*	34.7	27.3	40.8	47.7	41.4	0.8	0.0	24.9	37.7	0.7	0.0	20.8	42.8	2.9	0.0	15.7

## Data Availability

Not applicable.

## Supplementary Materials

The following supporting information can be downloaded at: https://www.mdpi.com/article/10.3390/antibiotics11070962/s1, Figure S1: Microbial metabolic activity over time for high lactulose treatment dose; Figure S2: Reciprocal Simpson diversity index for high lactulose treatment dose; Figure S3: Microbial community composition as assessed via 16S-targeted Illumina sequencing for high lactulose treatment dose; Table S1: Overall metabolic activity in terms of SCFA and lactic acid production for high lactulose treatment dose; Table S2: Microbial metabolic activity in terms of BCFA and ammonium production for high lactulose treatment dose; Table S3: Proximal microbial community composition as assessed via 16S-targeted Illumina sequencing at family level for high lactulose treatment dose; Table S4: Distal microbial community composition as assessed via 16S-targeted Illumina sequencing at family level for high lactulose treatment dose.
