# Supplementary material for: Co-Administration of Lactulose Crystals with Amoxicillin Followed by Prolonged Lactulose Treatment Promotes Recovery of the Human Gut Microbiome In Vitro
Source: Antibiotics (Basel). 2022 Jul 18;11(7):962. doi: 10.3390/antibiotics11070962 (PMC9312270; doi:10.3390/antibiotics11070962)
Supplement: Supplementary file 1 [file antibiotics-11-00962-s001.zip › antibiotics-1803346-supplementary.pdf]

Co-administration of lactulose crystals with amoxicillin followed by prolonged lactulose treatment promotes recovery of the human gut microbiome *in vitro*

Cindy Duysburgh [1], Pieter Van den Abbeele [1,#], Dennis Franckenstein [2], Martin Westphal [2], Angelika Kuchinka-Koch [3] and Massimo Marzorati [1,4,*]

Supplementary Materials

Figure S1. Microbial metabolic activity over time for high lactulose treatment dose. Absolute concentrations (mM) of acetate (a-b), propionate (c-d), butyrate (e-f) and lactate (g-h) in the proximal (PC) and distal colon (DC) reactors upon lactulose administration at a dose of 10 g/d during antibiotic treatment (LAC) as well as lactulose treatment during and prolonged following antibiotic treatment (PRL LAC) compared to an antibiotic control (AB CTRL) and a negative control (CTRL). Samples were taken during two control (C1 and C2), one antibiotic (AB) and two follow-up (FU1 and FU2) weeks. During each week, three samples were collected and shown separately in the graph.

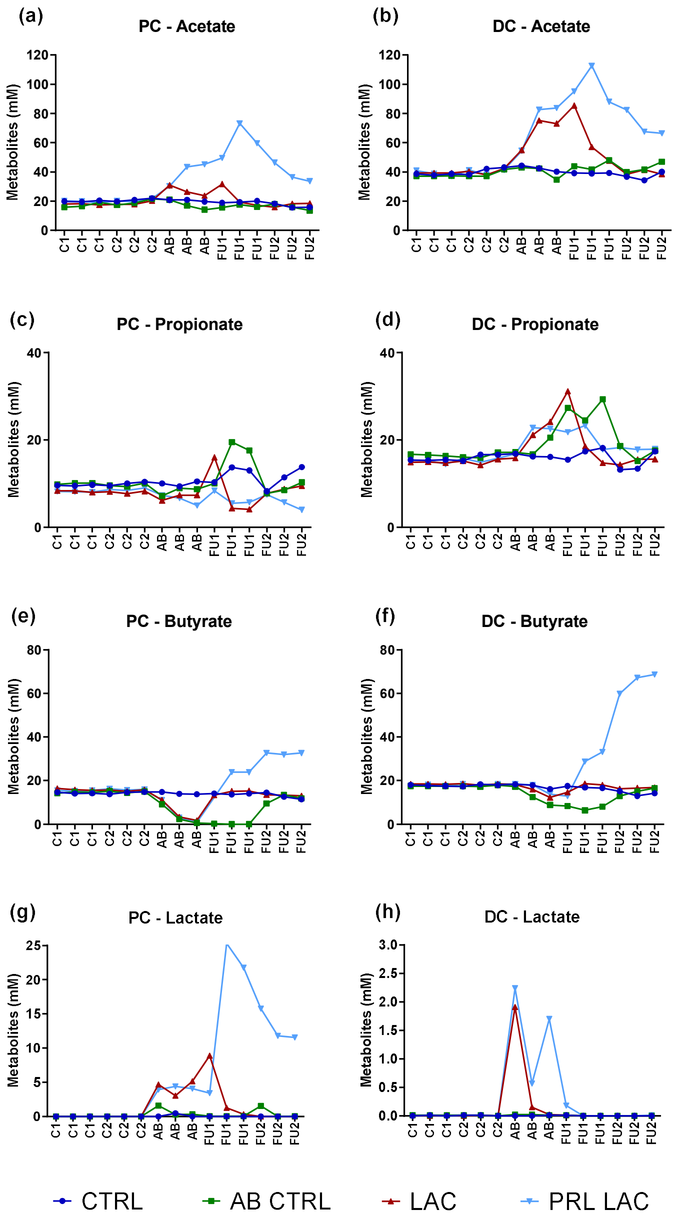

Figure S2. Reciprocal Simpson Diversity Index for high lactulose treatment dose. Reciprocal Simpson Diversity Index in the proximal (PC; a) and distal colon (DC; b) reactors at the end of the control period (C), antibiotic treatment (AB) and follow-up week 1 (FU1) and 2 (FU2) upon lactulose administration at a dose of 10 g/d during antibiotic treatment (LAC) as well as lactulose administration during and prolonged following antibiotic treatment (PRL LAC) compared to an antibiotic control (AB CTRL) and a negative control (CTRL) (n = 1).

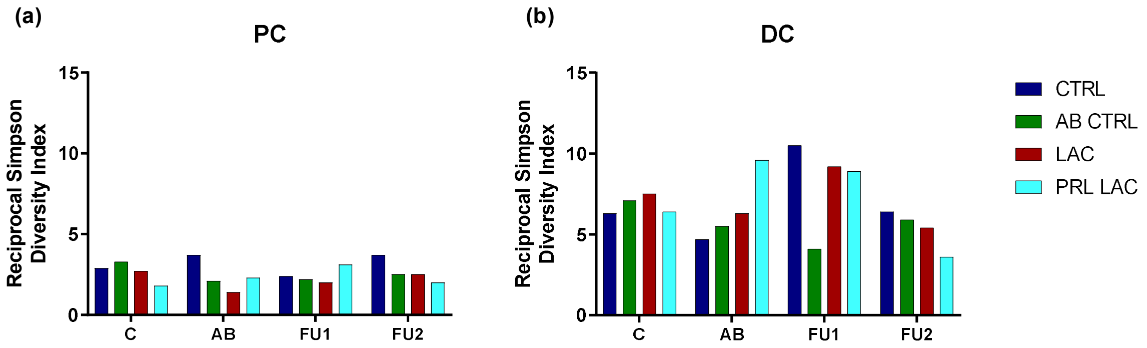

Figure S3. Microbial community composition as assessed via 16S-targeted Illumina sequencing for high lactulose treatment dose. Abundance (%) at microbial phylum level in the proximal (PC; a) and distal colon (DC; b) reactors at the end of the control period (C), antibiotic treatment (AB) and follow-up week 1 (FU1) and 2 (FU2) upon lactulose administration at a dose of 10 g/d during antibiotic treatment (LAC) as well as lactulose administration during and prolonged following antibiotic treatment (PRL LAC) compared to an antibiotic control (AB CTRL) and a negative control (CTRL) (n = 1).

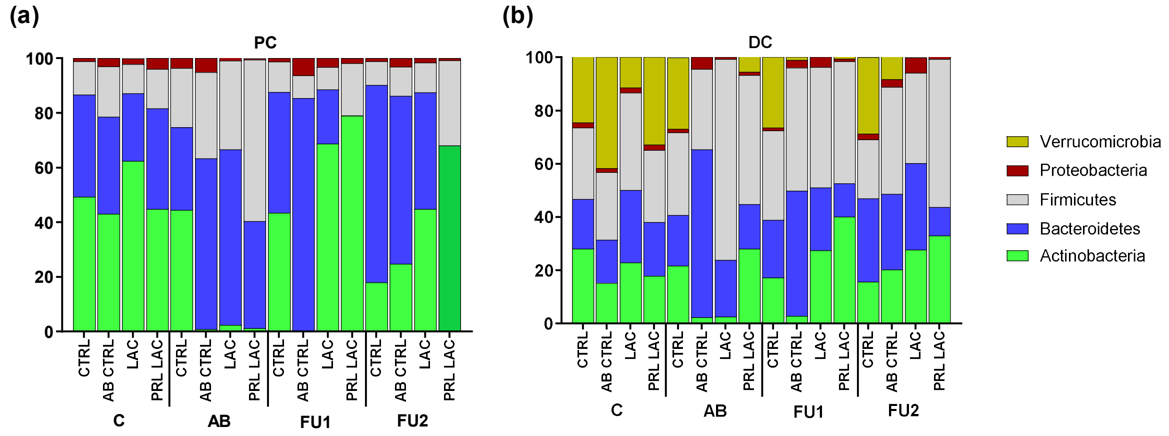

Table S1. Overall metabolic activity in terms of SCFA and lactic acid production for high lactulose treatment dose. Average acetate, propionate, butyrate and lactate production (mM) during the control (C; n = 6), the antibiotic (AB; n = 3) and the two follow-up (FU1/2; n = 3) weeks in the proximal (PC) and distal colon (DC) reactors upon lactulose administration at a dose of 10 g/d during antibiotic treatment (LAC) as well as lactulose administration during and prolonged following antibiotic treatment (PRL LAC) compared to an antibiotic control (AB CTRL) and a negative control (CTRL). Data is presented as mean ± stdev. Statistically significant differences relative to the control period are indicated in bold (p<0.05). The intensity of shading indicates the absolute concentration, normalized for each of the different metabolites per colonic region. Lowest values are indicated with two shades of red, medium values are indicated in white, whereas the highest values are indicated with two shades of green.

			C	AB	FU1	FU2
Acetate (mM)	PC	CTRL	20.4 (± 0.9)	20.5 (± 0.7)	19.5 (± 0.6)	**16.6** (± 1.5)
		AB CTRL	18.3 (± 2.0)	17.4 (± 3.4)	16.5 (± 1.1)	16.0 (± 2.2)
		LAC	18.3 (± 1.0)	**27.1** (± 3.6)	22.8 (± 7.8)	17.6 (± 1.5)
		PRL LAC	20.2 (± 0.9)	**39.7** (± 8.2)	**60.9** (± 11.9)	**38.9** (± 6.7)
	DC	CTRL	39.8 (± 2.3)	42.4 (± 2.1)	39.2 (± 0.1)	37.1 (± 2.9)
		AB CTRL	38.0 (± 1.8)	40.1 (± 4.6)	**44.5** (± 3.2)	42.9 (± 3.7)
		LAC	39.8 (± 1.5)	**67.8** (± 11.1)	63.4 (± 19.7)	39.5 (± 1.6)
		PRL LAC	39.7 (± 1.8)	**73.7** (± 16.6)	**98.7** (± 12.7)	**72.2** (± 9.0)
Propionate (mM)	PC	CTRL	9.8 (± 0.3)	9.9 (± 0.6)	12.3 (± 1.8)	11.1 (± 2.8)
		AB CTRL	9.8 (± 0.3)	**8.3** (± 1.0)	15.7 (± 5.0)	8.8 (± 1.3)
		LAC	8.1 (± 0.3)	**7.0** (± 0.7)	8.2 (± 6.8)	8.7 (± 0.9)
		PRL LAC	8.4 (± 0.4)	**6.3** (± 1.2)	6.5 (± 1.6)	5.7 (± 1.7)
	DC	CTRL	15.8 (± 0.7)	16.4 (± 0.4)	17.0 (± 1.4)	14.6 (± 2.3)
		AB CTRL	16.4 (± 0.4)	18.1 (± 2.1)	**27.1** (± 2.4)	17.1 (± 1.8)
		LAC	14.9 (± 0.4)	**20.4** (± 4.2)	21.5 (± 8.6)	15.1 (± 0.7)
		PRL LAC	15.3 (± 0.5)	**20.7** (± 3.4)	**21.0** (± 2.8)	**18.0** (± 0.2)
Butyrate (mM)	PC	CTRL	14.4 (± 0.4)	14.2 (± 0.5)	13.9 (± 0.3)	12.8 (± 1.6)
		AB CTRL	14.7 (± 0.4)	**4.0** (± 4.5)	**0.1** (± 0.1)	11.6 (± 2.0)
		LAC	15.7 (± 0.4)	**5.4** (± 5.0)	14.6 (± 1.1)	**13.3** (± 0.2)
		PRL LAC	15.7 (± 0.4)	**5.0** (± 5.4)	**23.9** (± 0.0)	**32.4** (± 0.4)
	DC	CTRL	17.8 (± 0.4)	17.4 (± 1.2)	17.0 (± 0.5)	**14.1** (± 1.1)
		AB CTRL	17.5 (± 0.2)	**12.9** (± 4.2)	**7.6** (± 1.0)	14.9 (± 1.8)
		LAC	18.4 (± 0.3)	**15.5** (± 3.0)	17.1 (± 2.1)	**16.6** (± 0.3)
		PRL LAC	18.0 (± 0.4)	16.8 (± 2.5)	24.9 (± 10.6)	**65.3** (± 4.7)
Lactate (mM)	PC	CTRL	0.00 (± 0.00)	0.16 (± 0.27)	0.01 (± 0.00)	0.00 (± 0.00)
		AB CTRL	0.00 (± 0.00)	**0.72** (± 0.75)	**0.05** (± 0.01)	0.53 (± 0.89)
		LAC	0.01 (± 0.00)	**4.28** (± 1.11)	3.48 (± 4.69)	0.00 (± 0.00)
		PRL LAC	0.00 (± 0.00)	**4.11** (± 0.24)	16.82 (± 11.77)	**13.04** (± 2.37)

	**DC**	**CTRL**	0.006	(±	0.004	)	0.004	(±	0.003	)	0.003	(±	0.005	)	**0.000**	(±	0.000	)
		**AB CTRL**	0.007	(±	0.004	)	0.016	(±	0.008	)	0.003	(±	0.005	)	**0.001**	(±	0.000	)
		**LAC**	0.004	(±	0.003	)	0.692	(±	1.055	)	0.003	(±	0.005	)	0.001	(±	0.001	)
		**PRL LAC**	0.004	(±	0.003	)	**1.503**	(±	0.853	)	0.061	(±	0.105	)	0.003	(±	0.003	)

Table S2. Microbial metabolic activity in terms of BCFA and ammonium production for high lactulose treatment dose. Average BCFA (mM) and ammonium (mg/L) production during the control (C; n = 6), the antibiotic (AB; n = 3) and the two follow-up (FU1/2; n = 3) weeks in the proximal (PC) and distal colon (DC) reactors upon lactulose administration at a dose of 10 g/d during antibiotic treatment (LAC) as well as lactulose administration during and prolonged following antibiotic treatment (PRL LAC) compared to an antibiotic control (AB CTRL) and a negative control (CTRL). Data is presented as mean ± stdev. Statistically significant differences relative to the control period are indicated in bold (p<0.05). The intensity of shading indicates the absolute concentration, normalized for each of the different metabolites per colonic region. Lowest values are indicated with two shades of red, medium values are indicated in white, whereas the highest values are indicated with two shades of green.

			C	AB	FU1	FU2
**bCFA (mM)**	**PC**	**CTRL**	2.73 (± 0.08)	2.72 (± 0.19)	2.76 (± 0.07)	2.57 (± 0.10)
		**AB CTRL**	2.74 (± 0.12)	**0.96** (± 0.89)	**0.31** (± 0.03)	2.62 (± 0.05)
		**LAC**	2.74 (± 0.05)	**0.88** (± 1.03)	2.49 (± 0.21)	2.55 (± 0.10)
		**PRL LAC**	2.78 (± 0.07)	**0.85** (± 1.04)	1.38 (± 1.04)	**1.87** (± 0.05)
	**DC**	**CTRL**	2.88 (± 0.08)	2.92 (± 0.28)	2.96 (± 0.09)	2.77 (± 0.17)
		**AB CTRL**	2.83 (± 0.07)	2.13 (± 0.84)	**1.16** (± 0.46)	2.76 (± 0.16)
		**LAC**	2.88 (± 0.07)	**1.22** (± 0.87)	1.94 (± 1.02)	2.74 (± 0.08)
		**PRL LAC**	2.88 (± 0.11)	**1.49** (± 1.18)	**0.91** (± 0.41)	**1.85** (± 0.02)
**Ammonium (mg/L)**	**PC**	**CTRL**	221 (± 19)	244 (± 12)	230 (± 22)	220 (± 19)
		**AB CTRL**	220 (± 15)	**124** (± 66)	**107** (± 4)	212 (± 34)
		**LAC**	217 (± 12)	**118** (± 63)	182 (± 22)	**248** (± 7)
		**PRL LAC**	234 (± 10)	**134** (± 57)	**117** (± 10)	**104** (± 8)
	**DC**	**CTRL**	345 (± 18)	332 (± 36)	359 (± 4)	371 (± 3)
		**AB CTRL**	336 (± 17)	304 (± 69)	**280** (± 32)	344 (± 18)
		**LAC**	340 (± 21)	**182** (± 111)	234 (± 117)	357 (± 21)
		**PRL LAC**	339 (± 14)	**185** (± 94)	**92** (± 23)	**100** (± 12)

Table S3. Proximal microbial community composition as assessed via 16S-targeted Illumina sequencing at family level for high lactulose treatment dose. Abundance (%) at microbial family level in the proximal colon (PC) reactor at the end of the control period (C), antibiotic treatment (AB) and follow-up week 1 (FU1) and 2 (FU2) upon lactulose administration at a dose of 10 g/d during antibiotic treatment (LAC) as well as lactulose administration during and prolonged following antibiotic treatment (PRL LAC) compared to an antibiotic control (AB CTRL) and a negative control (CTRL) (n = 1). The intensity of shading indicates the absolute abundance, normalized for each of the different families. Lowest values are indicated with two shades of red, medium values are indicated in white, whereas the highest values are indicated with two shades of green.

Phylum	Family	PC															
		C				AB				FU1				FU2			
		CTRL	AB CTRL	LAC	PRL LAC	CTRL	AB CTRL	LAC	PRL LAC	CTRL	AB CTRL	LAC	PRL LAC	CTRL	AB CTRL	LAC	PRL LAC
Actinobacteria	*Bifidobacteriaceae*	49.1	42.8	62.4	44.4	44.3	0.2	2.3	0.9	43.4	0.0	68.6	78.1	18.0	24.5	44.8	67.8
	Microbacteriaceae	0.0	0.1	0.0	0.4	0.1	0.7	0.0	0.3	0.0	0.1	0.1	0.7	0.0	0.3	0.0	0.2
Bacteroidetes	*Bacteroidaceae*	37.4	35.6	24.7	36.8	30.3	62.4	64.2	39.1	44.2	85.2	19.8	0.1	72.2	61.4	42.7	0.0
Firmicutes	*Acidaminococcaceae*	0.7	3.1	1.1	1.3	0.7	0.0	8.1	0.0	1.9	5.5	0.7	0.4	2.2	1.4	2.2	0.2
	Enterococcaceae	0.4	0.5	0.5	4.1	12.5	31.3	0.1	0.1	0.9	1.6	0.3	2.8	0.1	7.6	0.5	0.1
	Lachnospiraceae	9.0	9.1	6.5	6.7	7.1	0.0	8.1	49.6	6.8	1.2	2.0	0.5	5.4	1.1	6.0	13.6
	Lactobacillaceae	1.2	1.6	1.1	0.5	0.6	0.0	16.1	9.3	0.8	0.0	2.7	1.8	0.3	0.2	0.1	0.0
	Ruminococcaceae	0.0	0.0	0.0	0.0	0.0	0.0	0.0	0.0	0.0	0.0	0.0	0.0	0.0	0.0	0.0	0.0
	Veillonellaceae	0.9	4.0	1.5	1.8	0.7	0.0	0.1	0.0	0.7	0.0	2.5	13.6	0.6	0.4	2.1	17.3
Proteobacteria	*Burkholderiaceae*	0.3	0.5	0.2	0.4	0.2	0.2	0.0	0.0	0.3	0.0	0.5	0.7	0.2	0.1	0.2	0.5
	Desulfovibrionaceae	0.9	2.4	1.0	1.4	0.6	0.0	0.1	0.1	0.8	0.2	0.3	0.0	0.7	0.7	0.5	0.0
	Enterobacteriaceae	0.0	0.1	0.8	1.9	2.3	2.2	0.7	0.1	0.1	5.7	2.0	0.2	0.1	1.3	0.4	0.0
	Pseudomonadaceae	0.0	0.1	0.1	0.1	0.1	1.5	0.1	0.3	0.1	0.3	0.4	0.5	0.1	0.5	0.5	0.2
	Xanthomonadaceae	0.0	0.1	0.1	0.2	0.4	1.4	0.0	0.1	0.0	0.0	0.1	0.5	0.0	0.5	0.0	0.0

Table S4. Distal microbial community composition as assessed via 16S-targeted Illumina sequencing at family level for high lactulose treatment dose. Abundance (%) at microbial family level in the distal colon (DC) reactor at the end of the control period (C), antibiotic treatment (AB) and follow-up week 1 (FU1) and 2 (FU2) upon lactulose administration at a dose of 10 g/d during antibiotic treatment (LAC) as well as lactulose administration during and prolonged following antibiotic treatment (PRL LAC) compared to an antibiotic control (AB CTRL) and a negative control (CTRL) (n = 1). The intensity of shading indicates the absolute abundance, normalized for each of the different families. Lowest values are indicated with two shades of red, medium values are indicated in white, whereas the highest values are indicated with two shades of green.

Phylum	Family	DC															
		C				AB				FU1				FU2			
		CTRL	AB CTRL	LAC	PRL LAC	CTRL	AB CTRL	LAC	PRL LAC	CTRL	AB CTRL	LAC	PRL LAC	CTRL	AB CTRL	LAC	PRL LAC
Actinobacteria	*Bifidobacteriaceae*	27.9	15.2	22.9	17.6	21.7	1.7	2.4	27.9	17.2	2.6	27.4	39.8	15.6	20.2	27.7	32.5
	Coriobacteriaceae	0.0	0.0	0.0	0.1	0.0	0.0	0.0	0.0	0.0	0.0	0.1	0.2	0.0	0.0	0.0	0.5
	Microbacteriaceae	0.0	0.0	0.0	0.1	0.0	0.7	0.0	0.1	0.0	0.0	0.1	0.0	0.0	0.1	0.0	0.0
Bacteroidetes	*Bacteroidaceae*	14.1	9.8	17.5	12.3	15.7	52.9	15.8	9.3	18.3	45.5	12.7	2.2	28.7	21.9	24.5	3.0
	Prevotellaceae	1.4	2.3	2.0	2.0	1.8	8.2	1.2	2.3	2.2	0.7	3.0	0.7	1.5	2.4	2.4	0.3
	Rikenellaceae	0.0	0.0	0.0	0.0	0.0	0.0	0.2	0.3	0.1	0.0	0.0	6.7	0.0	0.1	0.0	5.4
	Tannerellaceae	3.1	4.1	7.6	5.8	1.5	1.8	4.0	4.8	1.2	0.9	7.7	2.8	1.0	4.0	5.5	2.0
Firmicutes	*Acidaminococcaceae*	0.6	0.7	0.4	0.7	1.1	0.5	1.3	0.5	0.8	3.1	1.4	0.2	1.0	2.0	1.5	0.1
	Enterococcaceae	0.0	0.0	0.1	0.4	1.7	5.6	0.0	0.0	0.1	0.2	0.0	0.3	0.0	0.5	0.1	0.0
	Lachnospiraceae	24.4	23.1	34.2	24.1	26.4	12.4	69.6	45.9	31.1	34.0	38.1	44.9	19.6	33.6	27.4	55.1
	Lactobacillaceae	0.2	0.1	0.1	0.1	0.3	0.0	0.4	1.4	0.0	0.0	2.1	0.1	0.1	0.1	0.1	0.0
	Ruminococcaceae	1.5	1.1	1.4	1.5	1.4	11.6	4.2	0.5	1.2	8.3	2.7	0.2	1.4	3.5	4.3	0.0
	Veillonellaceae	0.1	0.1	0.2	0.2	0.1	0.0	0.0	0.1	0.1	0.4	0.8	0.1	0.1	0.3	0.5	0.2
Proteobacteria	*Burkholderiaceae*	0.2	0.1	0.3	0.2	0.1	1.3	0.4	0.6	0.1	0.2	0.6	0.4	0.1	0.3	0.2	0.3
	Desulfovibrionaceae	1.5	1.2	1.4	1.4	0.8	1.5	0.2	0.3	0.7	1.6	2.3	0.5	0.4	1.3	1.1	0.3
	Enterobacteriaceae	0.0	0.0	0.0	0.1	0.1	0.5	0.0	0.0	0.0	0.6	0.0	0.0	0.0	0.0	0.1	0.0
	Pseudomonadaceae	0.1	0.1	0.1	0.2	0.2	0.5	0.0	0.1	0.2	0.3	0.1	0.1	0.2	0.6	0.2	0.0

	uncultured	0.1	0.0	0.0	0.1	0.0	0.0	0.0	0.1	0.0	0.1	0.7	0.0	1.4	0.7	4.2	0.1
**Verrucomicrobia**	***Akkermansiaceae***	24.7	41.8	11.5	32.9	26.9	0.0	0.0	5.7	26.5	1.1	0.0	0.7	28.8	8.4	0.0	0.0
